# A Review of Methods of Diagnosis and Complexity Analysis of Alzheimer's Disease Using EEG Signals

**DOI:** 10.1155/2021/5425569

**Published:** 2021-10-27

**Authors:** Mahshad Ouchani, Shahriar Gharibzadeh, Mahdieh Jamshidi, Morteza Amini

**Affiliations:** ^1^Institute for Cognitive and Brain Sciences, Shahid Beheshti University, Tehran, Iran; ^2^Shahid Beheshti University, Tehran, Iran; ^3^Institute for Cognitive Science Studies (ICSS), Tehran, Iran

## Abstract

This study will concentrate on recent research on EEG signals for Alzheimer's diagnosis, identifying and comparing key steps of EEG-based Alzheimer's disease (AD) detection, such as EEG signal acquisition, preprocessing function extraction, and classification methods. Furthermore, highlighting general approaches, variations, and agreement in the use of EEG identified shortcomings and guidelines for multiple experimental stages ranging from demographic characteristics to outcomes monitoring for future research. Two main targets have been defined based on the article's purpose: (1) discriminative (or detection), i.e., look for differences in EEG-based features across groups, such as MCI, moderate Alzheimer's disease, extreme Alzheimer's disease, other forms of dementia, and stable normal elderly controls; and (2) progression determination, i.e., look for correlations between EEG-based features and clinical markers linked to MCI-to-AD conversion and Alzheimer's disease intensity progression. Limitations mentioned in the reviewed papers were also gathered and explored in this study, with the goal of gaining a better understanding of the problems that need to be addressed in order to advance the use of EEG in Alzheimer's disease science.

## 1. Introduction

Alzheimer's disease (AD) is a neurological disease and is also the most common form of age-related dementia in today's culture. In 2018, it was reported that 50 million people globally have Alzheimer's disease. In 2030, this figure will be around 82 million, and in 2050, it will be around 152 million [1]. In recent decades, there has been a growing focus on using advanced electroencephalography (EEG) signal processing to predict or differentiate Alzheimer's disease. Neuroimaging studies have been extensively used to investigate the causes of AD and to increase the accuracy of AD diagnosis [2]. Since the brain is such a complex structure with complex nonlinear dynamics, complexity studies utilizing data from brain imaging such as EEG, magneto-encephalograms, and functional magnetic resonance imaging (fMRI) are becoming more common fMRI [3]. In several experiments, brain impulses from just one channel, such as an electrode in EEG, a channel in magneto-encephalograms, or a voxel in fMRI, were studied. The brain complexity waves have recently been utilized to help explain the complexity of AD disorders [4]. Sufficient research into brain imaging modalities may help to describe the pathways underlying AD and to provide valuable evidence for the diagnosis. Recently, some research has shown that degrees of difficulty may be used as biomarkers in the early detection of AD. There is currently no systematic study that reviews the various modulation techniques and discusses the complexity of AD brain disorders. Optimizing EEG analysis is important for designing low-cost, noninvasive wearable applications to screen Alzheimer's patients [5]. The choice of critical EEG channels may also aid in the development of new wearable technologies and the optimization of computing resources. Many experiments have looked at multiscale entropy (MSE) and MSE-based measurements of EEG signals from Alzheimer's patients [6].

MSE represents the degree of healthfulness of a biological process by its production physiologic signals through measuring the complexity of a physiologic time series over various time scales [5]. Many experiments have shown that the MSE values of EEG signals from normal persons are higher on small scales than those from AD patients, but lower on large scales than someone from AD patients [5, 7]. Furthermore, at broad scales, the slope of the MSE vs. size plot was observed to be higher for AD patients than for healthy controls. The EEG is a noninvasive experimental tool that shows how brain synapses work in real time. Quantitative EEG (qEEG) research offers many perspectives on EEG signals, including frequency, dynamic alterations, and source imaging. Various researches have explained that qEEG can diagnose the foregoing anomalies in AD patients [8]: (1) changes in EEG patterns, (2) decreased coordination, (3) diminished sophistication, and (4) neuromodulator defects as potential markers for brain activity assessment. Furthermore, qEEG offers objective and quantifiable data that can be replicated in subsequent trials, as well as the benefits of having less laboratory protocols and lower costs [8]. This makes it ideal for screening large-scale and early detection of AD. For the purpose, EEG has been thoroughly researched as a useful instrument for analyzing AD over the past few decades. Nonetheless, as far as we can see, few of the study findings assist physicians with their daily work or decision-making. The concern is that the EEG signal is noise-sensitive, with nonstationary properties, which makes detection difficult [9]. Furthermore, since there is so much variation between subjects, it is difficult to distinguish objects and patterns from natural brain function. Reliable biomarkers and rigorous diagnostic techniques that can derive valuable knowledge from jumbled EEG signals are also required urgently [9, 10].

For EEG signal processing, the wavelet transform has been suggested as an efficient tool for analyzing time and frequency. It entails convolving the EEG signal with a variable-width time window, and higher frequencies have narrower window widths, whereas lower frequencies have wider window widths. This adjusts well to the features of EEG signals, which are made up of short-duration high-frequency incidents and long-duration low-frequency incidents [11]. EEG pulse time-frequency measurement combined with machine learning (ML) methods could help with diagnosis and understanding of AD. Overfitting could be avoided by using machine learning algorithms like feature selection, which exclude data that is redundant from high-dimensional data [12]. The thesis is aimed at investigating robust functional biomarkers dependent on time-frequency features of qEEG and developing a computer-aided discriminant scheme for automatically classification EEG signals of AD and normal elderly controls (NC) as a result of the promising results obtained with the wavelet transform analysis and machine learning methods [13].

Many experiments have looked at multiscale entropy and MSE-based measurements of EEG signals from Alzheimer's patients. MSE represents the degree of healthfulness of a living process by its production physiologic signals through measuring the complexity of a physiologic time series over various time scales. Many experiments have shown that the MSE values of EEG signals from healthy controls are higher on small scales than those from AD patients, but lower on large scales than those from AD patients. Furthermore, at broad scales, the slope of the MSE vs. size plot was observed to be higher for AD patients than for healthy controls. Lately, machine learning techniques have been introduced to EEG research in order to enhance the recognition accuracy at AD patients of various severity levels, as well as stable subjects. Any MSE time scale could be used as a function in a machine learning algorithm [14]. For each EEG channel, Fan et al. [15] used 38 features for machine learning, including MSE features and other spectral and temporal features derived from the EEG data. A total of 24 EEG recordings were obtained from stable, mild, and severe AD patients. There were five binary and one ternary classification problem to solve. Fan et al. used 19 EEG channels to remove 380 MSE functionality [15]. Each channel's EEG signals contributed a series of 20 distinct MSE values calculated at scales 1–20. A total of 123 EEG recordings were obtained from stable people, people with very minor AD, people with mild AD, and people with moderate to serious AD.

## 2. Literature Review

**Figure 1:**
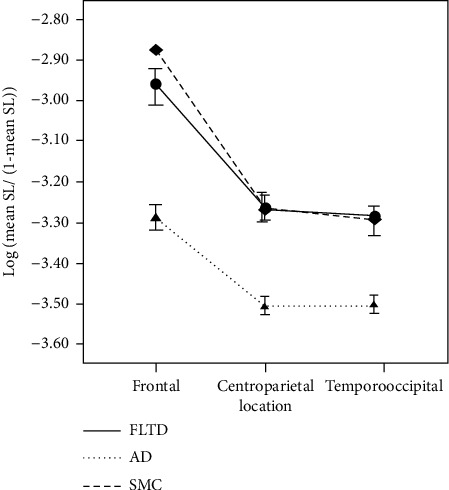
Electrode interaction effects caused significant group multiplication in the 8–10 Hz frequency range [20].

During rest, generalized EEG slowdown has been noticed in a variety of AD researches. This slowdown can be seen visually as a reduction in the dominant baseline rhythm's tempo, or spectrally as a rise in the strength of slow rhythms and a reduction in the power of quicker rhythms [16]. Indeed, in AD, the power spectrum's peak frequency is usually among 8-12 Hz variations to a lower range of 6–8 Hz. Just a few research has looked at EEG shifts in people with frontotemporal dementia. In frontotemporal dementia patients, qualitative examination of EEG recordings normally reveals no irregular slowing [17]. We would like to find out that pathological EEG slowdown is a more serious version of the general slowdown of the frame rhythm that occurs with healthy aging. As a result, age-matched control groups are needed in these studies; otherwise, the EEG-slowing effect would be exaggerated. A visual grand complete EEG score and the coordination probability as an indicator of functional connectivity were used to equate mild to moderate frontotemporal dementia and patients of Alzheimer's disease to healthy controls (HC) [18]. The complete EEG score in visual form did not vary significantly between frontotemporal dementia and HC. Using the visual grand total EEG, patients of Alzheimer's disease display substantial EEG slowing and lack of reactivity as compared to frontotemporal dementia and HC patients [19]. In high rates of frequency, AD patients have a lower chance of synchronization than frontotemporal dementia and HC patients, but there are no variations between frontotemporal dementia and HC patients (Figure 1). As a result, shifts in synchronization are likely to follow the slowing trend. Higher frequency features, such as strength and synchronization, are diminished in AD but not in frontotemporal dementia.

The qEEG variations are between people with frontal lobe dementia and others with Alzheimer's disease, Parkinson's disease dementia [21]. Lewy body disease has been studied in some trials. The global field power for six frequency bands was measured for the qEEG: *δ* (1 to 3.5 Hz), *θ* (4 to 7.5 Hz), *α* (8 to 11 Hz), *β*_1_ (12 to 15.5 Hz), *β*_2_ (16 to19.5 Hz), and *β*_3_ (20 to 23.5 Hz). The number of quick frequency bands was used to measure the spectral ratio *α* + *β*_1_ + *β*_2_ + *β*_3_ and bands of low frequency *δ* + *θ*. Patients with likely frontotemporal dementia were similar to AD patients and healthy controls on cortical EEG sources' spectral profile [22]. The authors of this study used EEG band forces, coherence, dominant frequency, peak frequency, and cortical sources to distinguish sixteen patients with AD from nineteen patients by frontotemporal dementia. The most accurate predictors of frontotemporal dementia and AD were identified in a model using logistic regression analysis. Activities such as elevated levels of visuospatial capacity and episodic memory were among the predictors. The model's classification accuracy was 93.3 percent.

**Figure 2:**
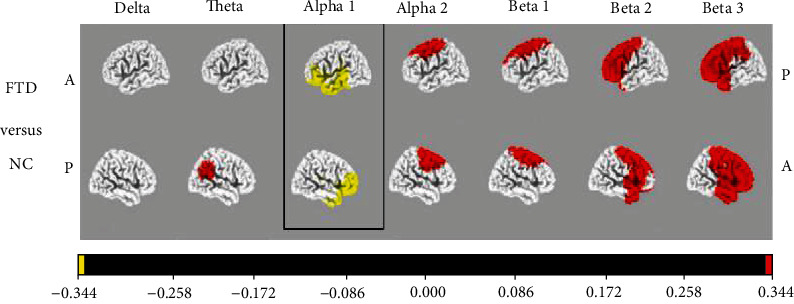
For the frontotemporal dementia and control classes, current density images in Talairach space collected by sLORETA were compared [25].

As a result, combining qEEG and neuropsychological assessments substantially improves classification performance and can be used for frontotemporal dementia and AD differential diagnoses [23]. Using power spectral analysis and uniform standardized low-resolution brain electromagnetic tomography within *δ*, *θ*, *α*_1_, *α*_2_, *β*_1_, *β*_2_, and *β*_3_, Caso et al. distinguished 39 Alzheimer's disease patients from among the frontotemporal dementia patients. As a result, the sensitivity is at the degree of chance. In comparison to HC, both studies showed higher expression of diffuse *δ*/*θ* and lower central/posterior quicker frequency bands in AD patients. Patients with frontotemporal dementia had diffusely higher *θ* capacity than HC patients and lower *δ* than AD patients. In comparison to frontotemporal dementia patients, AD patients had diffusely higher *θ* power in the power spectrum and reduced *α*_2_ and *β*_1_ in central/temporal areas using standardized low-resolution brain electromagnetic tomography. Slower frequencies are becoming more important, whereas higher frequencies are becoming less relevant. In patients with moderate levels of frontotemporal dementia and in HC, studies of global field force, which is a metric for the electric field pressure in the entire brain, were combined with EEG neuroimaging observations with low-resolution standardized brain electromagnetic tomography (sLORETA) [24]. Important group effects were found in the global field power in the *δ* (1.5 to 6 Hz), *α*_1_ (8.5 to 10 Hz), and *β*_1_ (12.5 to 18 Hz) bands. Differences in activation were seen in the 1 band (health control > frontotemporal dementia) in the orbital frontal and temporal lobes, the band (Alzheimer′s disease > health control) in widespread areas like the frontal lobe, and the *δ* band (frontal lobe dementia > Alzheimer′s disease) in the parietal lobe and sensorimotor region in low-resolution standardized brain electromagnetic tomography research (Figure 2). As a result, it does not appear that a particular brain area is essential in identifying these types.

**Figure 3:**
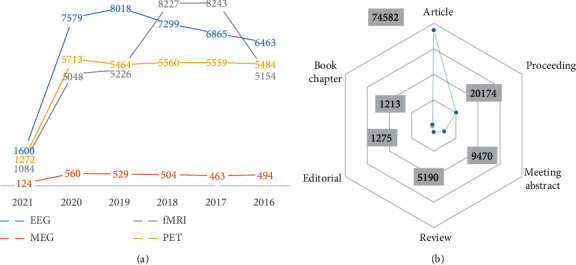
(a) Number of publications about EEG, EMG, fMRI, and PET between 2016 and 2021. (b) Types of published papers about EEG.

Snaedal et al. used qEEG to see whether they could tell the difference between 239 patients with AD, 52 patients to Parkinson disease, and 14 patients to FDT [26]. For grouping, the authors of this Icelandic analysis used *θ*, *α*_2_, and *β*_1_, as well as peak frequency. When utilizing a SVM method to classify cases of degenerative diseases from HC, a good-to-excellent distinction was observed, but this was less so when the risk of comorbidity was high [27]. The investigators were able to distinguish AD from Parkinson's disease dementia with 91 percent accuracy, 93 percent for Parkinson's disease dementia-frontotemporal dementia, and 88 percent for AD-frontotemporal dementia. Given the limited sample size of frontotemporal dementia patients, the precision of these statistical figures must be viewed with caution. In general, experiments including frontotemporal dementia face challenges in attracting volunteers, so the significance of this research should not be overlooked. Nonetheless, adequate feature subset selection is required for classification analysis, particularly in experiments with long vectors with features, such as this one, which included 1120 entries. It is unclear if the genetic algorithm's 10-fold cross-validation used a different preparation, assessment, and research collection in this analysis. This study reduced the original count of 382 studies to 126 studies after eliminating unqualified studies, as seen in Figures 3(a) and 3(b). EEG (64 percent), magneto-encephalograms (28 percent), and fMRI and practical near-infrared spectroscopy were the three types of studies (7%).

## 3. Preprocessing of EEG Signals

Hans Berger invented EEG, a noninvasive technique of functional imaging for studying the brain, in 1923. EEG measures the electrical output of a community of neurons to capture electric signals of the brain from the cerebral mantle [28]. EEG has a poorer spatial resolution than functional MRI but has a better temporal view into neuronal activity. Until operation EEG signals, five frequency bands are usually examined, *δ* (up to 4 Hz), *θ* (4 to 8 Hz), *α* (8 to 12 Hz), *β* (12 to 26 Hz), and *γ* (26 to 100 Hz) [29].  Table 1 summarizes these frequency bands and their associations with human activity.

EEG has a frequency range of 1–100 Hz and a voltage range of 10–100 *μ*V. To detect a disorder or decipher brain function using EEG data, utilizing the Fourier transform or wavelet transform, extract features and utilize spectral information from raw EEG dataset [29]. After that, the extracted features or transformed raw data are utilize to train a ML-based classifier, with deep learning algorithms proving to be effective at automated feature extraction for testing. Centered on the location of the reference electrode, EEG recording can be done in two ways [29].

Experts believe that picture preprocessing is a bad idea since it affects or alters the raw data's actual nature. Intelligent picture preprocessing, on the other hand, can give benefits and address issues, resulting in enhanced locally and globally feature recognition. Image preprocessing may have a significant beneficial impact on the quality of feature extraction and machine vision findings. The statistical normalizing of a data collection, which is a frequent step in many visual feature techniques, is comparable to image preprocessing [30]. This is why a thorough study of picture preprocessing is important. A local binary encoder utilizing gray scale data, for instance, will involve different preprocessing than a color SIFT method; moreover, some investigative effort may be necessary to fine-tune the picture preprocessing stage for optimal results. The pixel intensity measurements of point pairs are dealt with using local binary features. As a consequence, the evaluations are highly insensitive to lighting, brightness, and contrast, and picture preprocessing may not be required to get satisfactory findings. Current literature-based local binary pattern techniques do not generally require significant picture preprocessing; instead, they depend on a simple matching criterion that can be modified to accommodate for lighting or contrast [30]. A Fourier transform calculated across the whole picture or block is an example of a global or regional basis space feature that spans a regular-shaped polygon. However, basis space characteristics, such as the Fourier spectrum of the LBP summary, which may be computed over histogram bin values of a local identifier to give rotational normalization, may be part of the local features. Another case is the Fourier descriptor, which is used to construct polygon factors for radial line segment lengths to offer rotational invariance by displaying the roundness of a feature. Rather than fixing issues, enhancements are utilized to optimize for certain feature measuring techniques. Sharpening and color balance are two common picture processing improvements [30].

Harris hawk's optimization [31], multiswarm whale [32], Moth-flame optimizer [33–35], gray wolf [36, 37], fruit fly [38, 39], bacterial foraging optimization [40], boosted binary Harris hawk's optimizer [41], an1t colony [42, 43], biogeography-based whale optimization [44], and grasshopper optimizer [45] are some optimization methods based on metaheuristic algorithms. Furthermore, biological applications of machine learning are common, such as tuberculosis [46], thyroid nodules [47], Parkinson's disease [48], and paraquat-poisoned individuals [49, 50]. The reference electrode is located on an electrically inactive region, and the active electrode is located on an electrically active area (e.g., an ear lobe). Scalp EEG is the standard technique for capturing EEG signals, which involves placing electrodes on the surface of the skull [51]. The biggest disadvantage of scalp EEG is that due to the vast spacing among neurons within the skull and the electrodes, the captured signals become blurred. Intracranial electroencephalography signals are recorded by inserting electrodes on the exposed region of the brain to improve signal strength in terms of interference and amplitude [51].

## 4. Feature Extraction of EEG Signals

**Figure 4:**
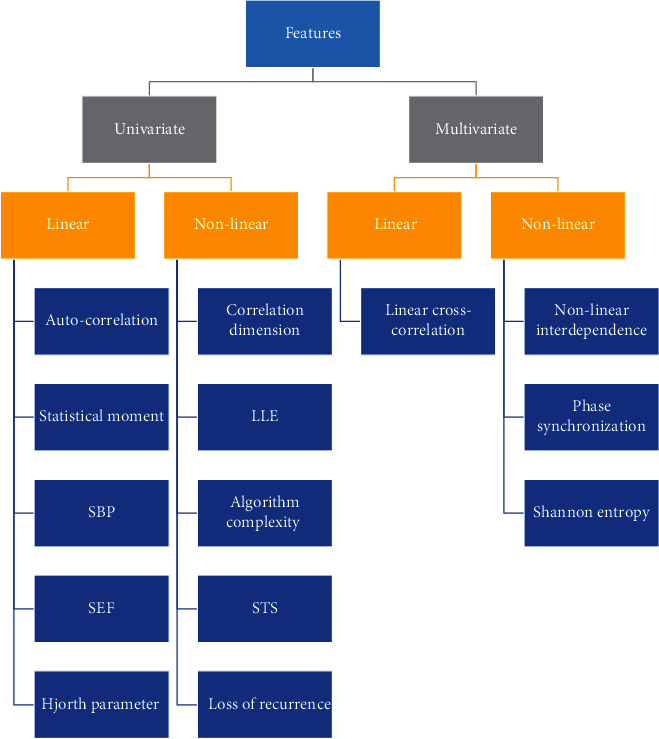
The number of channels in EEG data is used to categorize features.

Every prediction models must have consistent features that are well associated with the preictal and interictal levels. Those features may be classified as univariate (means that the measurements were taken separately on any EEG channel) or multivariate (means that the EEG measurements on two or up channels) on the basis of the amount of EEG channels. Of these may be further classified as linear or nonlinear elements. For ES estimation, Waser et al. contrasted the efficiency of univariate and bivariate tests that included methods that are both linear and nonlinear [52]. They discovered that preictal deviations occurred 5-30 minutes before the start of ES by using univariate tests. Bivariate tests, on the other hand, worked preferred by capturing preictal changes least 240 minutes afore the start of an ES.  Figure 4 depicts some of the linear and nonlinear ES estimation measures utilized in the related work. Nonlinear tests worked better or were equivalent to linear measures in some cases. Machine learning algorithms, such as artificial neural networks, *k*-means clustering, decision trees, SVM, and fuzzy logic, are used to identify preictal and interictal patterns from EEG results [53]. To draw conclusions, most people use thresholds based on function values. Machine learning-based research, on the other hand, has mostly focused on the processing of optimized features for projection. At the clinical stage, the EEG signal is provided in the couple the time and frequency domains. Since EEG signals are nonstationery and brain rhythms occur in time domain, also, the signal must be interpreted in both time and frequency domains [53] (see Figure 4).

**Figure 5:**
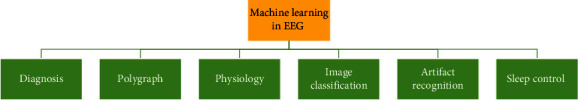
The process of EEG signal based on machine learning classifier.

The calculation of relative EEG power in each EEG frequency band is performed to check the slowing result in the EEG signal of Alzheimer's disease patients. Low-frequency bands (*δ* and *θ* bands), i.e., frequency area among 0.5 to 8 Hz, have a high relative power. The normative measure of EEG signal irregularity, such as Lempel Ziv complexity [54, 55], is used to quantify this irregularity. The spectrum of EEG signal is resulted by neurodegenerative disorders like mild cognitive impairment (MCI) and AD. Alzheimer's disease and mild cognitive impairment allow the EEG signal to slow down, according to recent research. The power in low-frequency bands (*δ* and *θ* bands, 0.5–8 Hz) is increased in EEG signals from Alzheimer's patients, while power in high-frequency bands (*α* and *β* bands, 8–30 Hz) is reduced. The power spectral density function aids in the evaluation of each epoch's spectral characteristics [56]. To achieve a normalized Postsynaptic density, also, the postsynaptic density is multiplied with the overall power in the frequency range of 0.1 to 40 Hz [56]. To acquire data from the EEG, good signal processing methods are needed because the data recorded by the EEG is a complex waveform. Doma and Pirouz [57] explained why the EEG signals are not stored in their normal state and why the captured data is not used for study in its original form. It is preferable to preprocess the EEG signals before beginning the process of extracting indications. The Fast Fourier Transform method is the most widely used signal processing method. Spectral, mapping, morphological localization, time metric, correlation, auxiliary, segment analysis, and other signal processing approaches should be noted.  Figure 5 depicts the use of neural networks in the area of EEG signal processing in this study.

Sadati et al. [58] used an adaptive diffusion neural network to diagnose epilepsy. Use DWT subband energy to extract features. However, their proposed method achieved an accuracy of about 85.9%. Ocak [59] proposed a method for feature extraction and DWT using approximate entropy and achieved an accuracy of more than 96% when using DWT and not using DWT. Nunes et al. did not just classify sentences A and E [60], but checked the complete data set of the University of Bonn (data sets A, B, C, D, and E) and checked various combinations of feature extraction and classification methods. The average accuracy of wood as a classifier is 89.2%. Subasi and Gursoy [61] studied various analysis methods to reduce the size of EEG data and combined EEG data with principal component analysis (PCA), linear discriminant analysis (LDA), and independent component analysis (ICA). Subasi [62] uses wavelet transform for feature extraction and expert model for classification. The overall accuracy of this method has reached 94.5%. Recently, Chen [63] introduced the double Fourier tree of complex waveforms as a feature extraction method and used the nearest neighbor classifier for classification. The proposed method achieved the ideal classification accuracy (100%). Djemili et al. used another newer method, which also achieved the desired classification speed. [64] uses empirical mode decomposition for feature extraction, and uses a multilayer perceptual neural network as a classifier.

## 5. Classification on Alzheimer Disease

EEG data are utilized to detect human brain diseases, as well as mental and emotional states, using a variety of deep learning architectures. The electroencephalogram (EEG) monitors the brain's neuro-activities, also known as brainwaves. Alpha waves, theta waves, beta waves, gamma waves, and delta waves are five distinct frequency waves. The neuroscience community has used several deep learning algorithms to analyze these brain waves in order to diagnose brain diseases and recognize human feelings [65]. Convolutional neural network (CNN), auto encoder (AE), recurrent neural network (RNN), deep belief network (DBN), restricted Boltzmann machine (RBM), multilayer perceptron neural network (MLPNN), optimized deep neural network, and EEG-functional magnetic resonance imaging- (EEGfMRI-) based deep learning are some of the most common learning algorithms.

Most classification trials, to our knowledge, have used data obtained from healthy people [66]. EEG data was used in 27 of these experiments to characterize feelings. Since our method uses a four-electrode EEG sensor, we will concentrate on studies that have used a small number of electrodes. Past experiments that used EEG data from up to four electrodes connected to healthy individuals are summarized in Table 1. Seo et al. [67, 68] and Kim et al. [69] used EEG data obtained from two electrodes. Lee et al. [70], for example, learned an SVM model but did not disclose the model's accuracy. Furthermore, rather than teaching a model, Kim et al. used general research to examine the association between EEG and eye-tracking data [69]. Lee et al. [70] did not go into depth about the methods they used for model output validation (if any). Finally, 5-fold cross-validation and leave-one-out cross-validation were used by Seo et al. [67, 68] to test their models. The experiments in Table 2 are aimed at classifying the feelings of healthy people; as a result, their findings may not be specific to patients with neurological disorders. However, based on these findings, we can infer that EEG data collected from electrodes on the forehead has the capacity to distinguish human emotions. As a result, classifying emotions using EEG data taken from an AD patient's forehead may be a promising avenue to pursue.

The association among signals *x* and *y* as a frequency structure, varying from 0 to 1, is known as coherence. Volume conduction through the scalp can have an effect on this measurement. In two trials, *θ* range coherence was found to be stronger in than in AD [54, 55]. In one study [74], *α* and *β* coherence were shown to be lower in dementia with Lewy body disease relative to AD, whereas other studies [75] found higher *α* and *β* coherence in dementia with Lewy body disease. Granger causality is also utilizing to describe how the time course of the EEG in channel *X* could be used to estimate possible EEG signal values in channel *Y*. According to one study, parietal area Granger causality is slightly greater in dementia with Lewy body disease than in AD, with a high precision of ~100%. The PLI calculates a stable causal delay among two signal sources and is slowly influenced with volume conduction on the scalp. PLI ratings range from 0 to 1, with 0 indicating no causal synchronization and 1 indicating complete causal synchronization. Dementia with Lewy body disease had a lower PLI within the *α*  spectrum than AD, suggesting more extreme improvements in connectivity in dementia with Lewy body disease. The changes in *α* network connectivity are consistent with another analysis that found lower mean *α* band guided phase shift entropy in dementia with Lewy body disease relative to AD, which tests posterior-to-anterior connectivity [76].

Weighted phase lag index (PLI) is a variation of phase lag index that entails weighting the PLI rates by the imaginary portion of the cross-spectrum between the two time-series [77]; the latter part of the cross-spectrum is related to the phase difference, or delay, between the signals. The two signs are nearly overlapping if the imaginary component is close to 0. One advantage of weighted PLI can be significantly raised by loud conducting sources, although this effect is less pronounced in weighted PLI [78]. Only one study used this method and found that dementia with Lewy body disease had a lower weighted PLI in the *β* band than Alzheimer's disease [79]. LLC is a connectivity metric that is calculated with the aid of precise low-resolution brain electromagnetic tomography tools. LLC is less affected by volume conduction and calculates functional cortical source connectivity by eliminating zero-lag instantaneous step coupling among cortical sources of resting state EEG rhythms. When comparing AD to dementia with Lewy body disease, LLC in the *α* and *δ* levels was lower in AD, which Babiloni et al. [22] speculated may indicate that AD had more cortical disconnection as both disorders progressed to dementia [22]. To test functional network connectivity, a graph technique focused on weighted network, and least spanning tree (MST) processes was used. The one study that looked at weighted PLI found that dementia with Lewy body disease (LBD) had lower connectivity and more network segregation in the *β* network than AD [80]. MST was used in four experiments [81, 82], all of which found that dementia with LBD had a less degree, less Euclidean distance, upper diameter, higher eccentricity, and less leaf-fraction than AD [82], implying a less-efficient network. Lewy body disease tends to have a randomized sequence consistent with decreased performance and synchronization [82]. EEG connectivity results in dementia with Lewy body disease (LBD) are summarized in Table 3.

## 6. EEG Signal Complexity Analysis of AD

A variety of nonlinear approaches have been used to investigate the features of brain function in Alzheimer's patients, yielding a host of intriguing findings. Resting-state recordings offer more accurate estimates of brain adaptability because they are not affected with task-specific arousal or discrepancy in impetus or success [84]. Resting brain function records and task-related observations show network dynamics that are close [85, 86] and also represent the influence of metabolically active networks. The time resolution of the EEG signal is very high, and it has been discovered that the signals have been studied mostly in various frequency bands and using from electrodes to show the diversity in signal rates.

### 6.1. The Signal Complexity Analysis in EEG

The signal complexity of the resting-state EEG in spinal cord injury, MCI, and AD patients is compared to standard controls in this segment. As applied to EEG signals, multiplex complexity technique, such as LZC, entropy complexity, and another complexity characteristics, has been shown to vary between spinal cord injury, MCI, AD, and control subjects in many experiments. Hogan et al. [87] discovered that MCI patients had a low entropy. According to a new analysis, the difficulty rates of EEG signals from AD patients are lower than those of spinal cord injury patients in all channels. ApEn and SampEn [3] in EEG signals have been seen to be slightly lower in healthy control and Alzheimer's disease patients relative to healthy individuals [88]; Garn et al. used various approaches [89] to investigate the complexity of EEG signals from AD patients and maturity clinical trial. In the EEGs of patients with AD, consistent findings were observed, including a substantial decrease in complexity at electrodes P_3_, P_4_, O_1_, and O_2_ positioned over the parietal, occipital, and temporal areas as compared with the healthy people. The medial temporal lobe, which is linked to short-term memory, is impaired during the MCI stage, as are the lateral temporal lobe and parietal lobe. The frontal lobe is compromised in the moderate stage of Alzheimer's disease. The occipital lobe is compromised during the acute stage of Alzheimer's disease [90]. The brain states that form during the transition from safe to AD have been studied using a variety of entropy approaches. The majority of the research has concentrated on specific regions of the brain. Patients with Alzheimer's disease and healthy control have less En values in all five areas (EnAD EnMCI EnControl), with major variations in the frontal, temporal, and central areas. These findings indicate that the frontal, temporal, and central EEG impulses in AD and MCI patients' brains were slightly less complex than those in HC. Furthermore, AD patients have the least difficulty and the most consistency. The complexity of EEG signals declines with disease progression, as predicted, particularly for comparing HC issues to Alzheimer's disease patients [91].

### 6.2. Conditions of EEG Recording and Symptoms of AD

Many experiments have looked at the impact of AD and its development on EEG signals over the past few decades. EEG signals have been used in studies under a variety of recording environments, which can be divided into two categories:

#### 6.2.1. Resting State

The brain background activation is measured by recording spontaneous EEG activity in the absence of some sort of stimuli. The acquisition of EEG data becomes less difficult, rather relaxed, and less stressful for the user, particularly for aged people [92], since the person is not expected to perform any particular task. A condition of rest EEG records include recordings made while resting-awake as well as recordings made while sleeping. AD has been shown to have four distinct impacts on resting-state EEG signals:

1. *Slowing*. In AD patients, power spectrum transitions from up-frequency ingredients (*α*, *β*, and *γ*) to low-frequency components (*δ* and *θ*) are normal [14, 93, 94]. The lack of cholinergic innervations in AD patients is believed to be the cause of this transition, which is proportional to the progression of the disease. The slowing of the EEG has been quantified using features obtained from the power curve, power spectrogram, and wavelet analysis
2. *Reduced Complexity*. By comparing AD patients to healthy controls, a reduction in the complexity of brain electrical activity has been found [14, 94–96]. Massive neuronal death and decreased interactions in cortical regions are likely to blame for this decline, which results in simplified EEG dynamics. Entropy metrics, auto mutual detail, Lempel-Ziv complexity, fractal dimension, and the Lyapunov exponent are some of the signal processing techniques used to investigate the complexity of EEG signals [92]
3. *Synchronization Declines*. This has been seen in many AD patients as a decrease in communication between cortical regions. While the cause of this syndrome is unknown, it is believed to be linked to atrophy in neural network connectivity
4. *Deficiencies in Neuromodulation*. Via cross-frequency interaction effects, the utilization of amplitude modulation to test EEG rhythms and brain neuromodulatory acting has lately been proposed [92]

### 6.3. EEG Recordings Associated with a Particular Event

The signals from the EEG are time-blocked, meaning they are captured in response to the occurrence of a single event. EEG operation is step locked as well as time-blocked, earning it the term event-related potentials. Induced activity is described as EEG activity that is not phase locked and can be examined using either event-related (de) synchronization [23] or event-related oscillations. Sensorial perceptive, motor, and cognitive functions can all be linked to events. Recent studies of the utilized event-connected EEG for Alzheimer's disease detection have been published in the AD literature. Although event-connected EEG studying enables researchers to investigate the impact of AD on individual brain circuits, these monitoring environments are not suitable for most AD patients, who experience a rise in anxiety and frustration, as well as a decline in their ability to do new things, even in the early stages of the disease. As a result, even completing a basic memory task may cause the patient pain and anxiety; they can become disoriented or unable to accomplish it [97]. Resting-state protocols, also, do not include extraneous stimulation, making them more straightforward and convenient for patients. Furthermore, these protocols produce less artifacts.

Some new articles on resting-state research for Alzheimer's disease detection have also been published. None of them, however, have focused solely on the subject of EEG-regarding Alzheimer's disease detection. Some reviews, for example, do not look at EEG as a primary diagnostic tool [93, 94, 98], whereas others are solely concerned with EEG signal synchronization [95, 96]. Furthermore, other publications [99, 100] offer a wider overview of the entire dementia continuum, not just AD. In revisions [101, 102], the key function types for Alzheimer's disease detection are thoroughly explored. As a result, the current research adds to previous studies of EEG-regarding Alzheimer's disease detection by regularly and exclusively analyzing papers on resting-state EEG to offer a comprehensive overview of the current state of the subjects.

## 7. Datasets

Three styles of datasets had been used for enforcing and verifying our methods. The first datasets are used for epilepsy diagnosis, and the 1/3 is used for autism diagnosis. The first dataset is provided through the Bonn University, Germany, and protected 5 units, named A, B, C, D, and E. Each set includes precisely a hundred single-channel EEG signals. Sets A and B had been accrued from scalp EEGs of neurotypical persons, while units C, D, and E had been accrued the use of intracranial EEGs from epileptic persons. The general duration of every sign is about 23.6 s. The records had been accrued with a sampling frequency of 173.61 Hz. The reference furnished in [103] indicates an extra specified description of this dataset. The study crew from MIT, USA [104], affords the second one dataset, which incorporates 906 h of EEG records accrued from 23 epileptic patients. In this study, handiest records for the primary twelve epileptic topics had been used, in conjunction with the ones of 11 neurotypical topics. This record consists of 23 EEG channels with a sampling frequency of 256 Hz [105].

The 1/3 dataset become furnished with the aid of using King Abdulaziz University (KAU) Brain–Computer Interface (BCI) Group, Jeddah, Saudi Arabia. The dataset become accumulated in a comfortable kingdom and cut up into groups: the primary institution become named the neurotypical institution and protected information from ten healthful volunteer subjects (all men, age 9–sixteen years) with common intelligence and with none intellectual disorders. The 2nd institution become classified the autistic institution and protected 9 subjects (six men and 3 females, elderly 10–sixteen years) with ASD. The EEG indicators had been accumulated from the subjects' scalps in a comfortable kingdom the usage of an EEG information-acquisition machine that protected the subsequent components: a g.tec EEG cap with excessive accuracy, sixteen Ag/AgCl sensors (electrodes) primarily based totally at the 10–20 global acquisition machine, g.tec USB amplifiers (gtec scientific engineering company, Schiedlberg, Austria), and BCI2000 software (The Brain-Computer Interface R&D Program on the Wadsworth Center of the New York State Department of Health in Albany, NY, USA). The dataset become filtered with the aid of using a band-byskip clear out with a passband of 0.1–60 Hz, and a notch clear out become used with a stopband frequency of 60 Hz. All EEG indicators had been digitized at a sampling frequency of 256 Hz. The EEG series time ranged from 12 to forty min for autistic sufferers with a complete of as much as 173 min. For neurotypical sufferers, the recording is various among five and 27 min with a complete of as much as 148 min.

## 8. Prodromal Dementia with LBD

Even at the mild cognitive impairment level, EEG anomalies on visual rating have been noted to be more frequent in dementia with LBD. When comparing MCI with Lewy bodies (MCI-LB) to MCI due to AD (MCI-AD), MCI-LB had more diffused anomalies (76 percent vs. 8%) and FIRDA (22 percent vs. 0%) [106]. EEG severity ratings were also slightly lower in MCI-LB, with just 16 percent of MCI-LBs having regular EEGs compared to 49 percent of MCI-ADs [106]. MCI-LB empirical EEG results have been compared to those published in dementia with Lewy body disease and Alzheimer's disease, with MCI-LB having a lower dominant frequency than MCI-AD [106, 107]. This results in a higher *θ*/*α* ratio and higher pre-*α* power, as well as lower *α* and *β* power and a lower *θ*/*α* ratio [106–108]. In identifying MCI-LB (Table 4), Schumacher et al. 2020b found that spectral strength tests had sensitivities of 23 to 51 percent, precision of 81 to 97 percent, and a region under receiver operating specification curve of 0.54 to 0.71 [108]. Van der Zande et al. 2020, but on the other hand, registered an AUROC of 0.76 to 0.97, but no sensitivities or specificities [106]. The MCI-LB connection was only studied in one study, which showed that LLC was lower in MCI-LB and MCI-AD as compared to age-matched controls, but no difference between the MCI groups [22] (see Table 4).

Several research looked at whether EEG features would predict dementia development in MCI patients. In one study, MCI patients who progressed to dementia with Lewy body disease (MCI-LB) had a lower mean frequency and *α*/*θ* ratio than those who suggested MCI-AD [107]. Other research utilized CSA to assess progression from MCI to dementia with Lewy body disease, AD, or no progression at 3 years in patients by MCI, with an average accuracy of 76%. Both patients with MCI who progressed to dementia with Lewy body disease had a CSA pattern of >1 (1-5) at baseline, while 93 percent of patients who improved to Alzheimer's disease had a CSA pattern of 1 (stable*α*) at baseline [111]. However, in 75% of patients with MCI, the involvement of one or more central or positive clinical characteristics of dementia with Lewy body disease predicted progression to dementia with Lewy body disease. The dominant frequency variability was comparable when MCI patients who advanced to dementia with Lewy body disease (MCI-LB) were compared to dementia with Lewy body disease patients, despite dementia with Lewy body disease patients having lower mean dominant frequencies [111]. After having comparable MMSE scores at baseline and a similar decrease on follow-up (20.6 in dementia with Lewy body disease and 20.5 in Alzheimer's disease), follow-up EEG of MCI-dementia with LBD patient's demonstrated improvement, with all patients with CSA 1 plus progressing to CSA 2 or 3. In contrast, considering the cognitive impairment, follow-up EEG of patients with MCI-AD revealed no progression (93 percent with CSA trend 1).

## 9. Conclusions

Alzheimer's disease is a sophisticated brain disease with massive financial, social, and medical consequences. It is recognized as the leading cause of dementia, characterized by amyloid peptide and phosphorylated tau (p-tau) protein accumulation and aggregation, as well as dementia, neuron loss, and brain atrophy. Despite decades of study, no acceptable medication exists that will stop the progression of Alzheimer's disease by acting on the illness's root cause, whereas currently existing therapies merely give symptomatic relief and do not provide a definitive cure or protection. Clinical signs, health information, family consultations, and current screening procedures such as clinical, neurological, and psychiatric examinations are used to diagnose Alzheimer's disease, whereas neuropsychological testing can be acknowledged as a tool for detecting unbiased signs of memory disturbances in the early stages, and laboratory studies such as thyroid function tests and serum vitamin B12 are used. To wrap up this report, we will discuss some of the remaining problems and study topics. Obtaining EEG data from MCI or AD patients is currently very complicated. In comparison to ECG and other biomedical records, such databases are not open to the public. As a consequence, consistently benchmarking and evaluating the latest approaches for the detection of Alzheimer's disease from EEG signals are difficult. Furthermore, almost none of those techniques integrate biophysical information about AD; comprehensive mathematical models of AD pathology combined with EEG data analysis can aid in improving AD diagnosis. Combining EEG with another signal and imaging methods, such as MRI dMRI, TMS, and SPECT, may yield even better results. The relationship among AD risk criteria (e.g., elevated homocysteine levels in the blood) and EEG characteristics needs to be studied further. Furthermore, the exact relationship between cognitive and memory loss and EEG disorders in Alzheimer's patients is still largely unknown. It is also crucial to see how EEG can help differentiate between MCI and various phases of AD, as well as between AD and other dementias. The EEG monitoring state is an important degree of freedom: it may be recorded (i) when the subject is at rest; (ii) when the subject is performing working-memory or other tasks; and (iii) when the subject is being activated with auditory, visual, tactile, or other cues. EEG signals can be more or less discriminative for MCI and AD depending on the recording situation; a thorough exploration of various recording situations with the goal of detecting MCI and AD is needed. In the future, it is also essential to evaluate the EEG in clinical studies of Alzheimer's disease, where the disease's development can be closely monitored; such studies may help us relate EEG abnormalities to AD neuropathology. Another intriguing line of investigation is the effect of treatment and therapy on the EEG of AD patients.

## Figures and Tables

**Table 1:** Frequency bands in EEG and associated studies of brain control [29].

Bands	Range (Hz)	Human nature and the relationship
*δ*	1-4	Infants and average adults' deep sleep periods are the most common places to see it.
*θ*	4-8	A high *θ* rhyme meaning in awake adults indicates irregular cognitive function.
	8-12	In normal relaxed people, it is usually found in the posterior area of the brain.
*β*	12-26	Present in the frontal lobe of the brain and in nervous people who are conscious.
*γ*	26-1000	Predominantly present in people who are anxious, satisfied, or conscious.

**Table 2:** EEG data are used to classify the emotions of healthier individuals (up to four electrodes).

Investigation	Emotional responses to be targeted	Method	Accuracy	Test
[71]	Happiness, rage, sorrow, fear, relaxation	Support vector machine (SVM)	73.32	Leave-one-out cross-testing
[72]	Engagement, perplexity, dissatisfaction, positive attitude	SVM, *k*-nearest neighbors (KNN)	95.69	—
[73]	Sorrow, displeasure	Multiclass support vector machine classifier	84.83	—
[70]	Arousal, sensitivity	SVM, *K*-means	—	—
[67]	Dissatisfaction, satisfaction	KNN	86.73	5-fold cross-testing
[68]	Dissatisfaction, satisfaction	Multilayer perceptron (MLP)	79.98	5-fold cross-testing
[69]	Boredom, frustration	Analysis	—	—

**Table 3:** Lewy body disease, a review of studies on EEG connectivity controls.

Author	Subband	Metrics	Outcome
[82]	*β*	Phase transfer entropy	AD > LBD
[80]	*β*	Weighted phase lag index	AD > LBD
[81]	*α*	Phase lag index	AD > LBD
[83]	*α*	Phase lag index	AD > LBD
[81]	*α*	Phase lag index	AD > LBD
[83]	*α*	Phase transfer entropy	AD < LBD
[22]	*α*	Lagged linear connectivity	AD < LBD/Parkinson′s disease dementia
[22]	*δ*	Lagged linear connectivity	AD < LBD/Parkinson′s disease dementia

**Table 4:** Basic EEG features' classification accuracy.

EEG features	Studies	TPR	FPR	ACC	AUC
Dementia with Lewy bodies vs. AD	[81]	97%	100%	99%	—
EEG severity grade	[109]	72–79%	76–85%	—	0.78–0.90
Grand total EEG	[22] [100]	65–78%	67–74%	70–73%	0.72–0.75
Occipital *α* power	[22]	78%	67%	73%	0.72
*δ* standard deviation	[110]	92%	83%	—	0.94
*θ* FP + *θ* power + *θ* − *α* DFV	[111]	~100%	~100%	~100%	—
Combined spectral array pattern	[83]	93%	97%	95%	—
Phase lag index *β* band	[82]	80%	85%		0.86
Minimum spanning tree-phase lag index	[80]	47%	100%	66%	0.78
P300- reversed amplitude distribution gradients	[21] [80] [112] [113]	76–100%	77–100%	66–100%	0.78–0.93
Machine learning algorithms	[106]	—	—	—	
EEG severity grade > 2	[106]	—	—	—	0.76
Diffuse abnormalities	[106] [108]	51%	86%	—	0.84
Peak/dominant frequency	[106] [108]	61%	81%	—	0.70–0.89
*β* power	[106] [108]	41%	97%	—	0.71–0.91
*α* power	[108]	56%	83%	—	0.66–0.85
Pre-*α* power	[106] [108]	33%	89%	—	0.68
*θ* power	[106] [108]	23%	89%	—	0.60–0.94
*δ* power	[106] [108]	49%	83%	—	0.54–0.55
*θ*/*α* ratio	[106]	—	—	—	0.64–0.92

## Data Availability

This is a review paper and data is not applicable.
